# Human Fetal Testis Xenografts Are Resistant to Phthalate-Induced Endocrine Disruption

**DOI:** 10.1289/ehp.1104711

**Published:** 2012-04-17

**Authors:** Nicholas E Heger, Susan J Hall, Moses A Sandrof, Elizabeth V McDonnell, Janan B Hensley, Erin N McDowell, Kayla A Martin, Kevin W Gaido, Kamin J Johnson, Kim Boekelheide

**Affiliations:** 1 Department of Pathology and Laboratory Medicine, Brown University, Providence, Rhode Island, USA; 2 The Hamner Institutes for Health Sciences, Research Triangle Park, North Carolina, USA; 3 Alfred I. duPont Hospital for Children, Wilmington, Delaware, USA; 4 Food and Drug Administration, Center for Veterinary Medicine, Rockville, Maryland, USA

**Keywords:** animal model, fetal testis, human, mouse, multinucleated germ cells, phthalate, rat, seminiferous cords, testicular dysgenesis, xenotransplant

## Abstract

Background: *In utero* exposure to endocrine-disrupting chemicals may contribute to testicular dysgenesis syndrome (TDS), a proposed constellation of increasingly common male reproductive tract abnormalities (including hypospadias, cryptorchidism, hypospermatogenesis, and testicular cancer). Male rats exposed *in utero* to certain phthalate plasticizers exhibit multinucleated germ cell (MNG) induction and suppressed steroidogenic gene expression and testosterone production in the fetal testis, causing TDS-consistent effects of hypospadias and cryptorchidism. Mice exposed to phthalates *in utero* exhibit MNG induction only. This disparity in response demonstrates a species-specific sensitivity to phthalate-induced suppression of fetal Leydig cell steroidogenesis. Importantly, *ex vivo* phthalate exposure of the fetal testis does not recapitulate the species-specific endocrine disruption, demonstrating the need for a new bioassay to assess the human response to phthalates.

Objectives: In this study, we aimed to develop and validate a rat and mouse testis xenograft bioassay of phthalate exposure and examine the human fetal testis response.

Methods: Fetal rat, mouse, and human testes were xenografted into immunodeficient rodent hosts, and hosts were gavaged with a range of phthalate doses over multiple days. Xenografts were harvested and assessed for histopathology and steroidogenic end points.

Results: Consistent with the *in utero* response, phthalate exposure induced MNG formation in rat and mouse xenografts, but only rats exhibited suppressed steroidogenesis. Across a range of doses, human fetal testis xenografts exhibited MNG induction but were resistant to suppression of steroidogenic gene expression.

Conclusions: Phthalate exposure of grafted human fetal testis altered fetal germ cells but did not reduce expression of genes that regulate fetal testosterone biosynthesis.

Recent adverse trends in male reproductive health, with an increase in both congenital malformations (hypospadias, cryptorchidism) and adult onset diseases (lowered sperm counts, testis germ cell cancer) have led to a proposed “testicular dysgenesis syndrome” (TDS) (Skakkebaek et al. 2001). The manifestations of TDS are thought to result from perturbations to fetal testis development, altering fetal testis hormone production and causing subsequent reproductive malformation and disease (Sharpe and Skakkebaek 2008). Recently, endocrine-disrupting chemicals (EDCs) such as phthalate esters have been associated with the development of TDS (Fisher 2004; Fisher et al. 2003; Hutchison et al. 2008), as rats exposed to phthalates *in utero* exhibit a similar array of male reproductive tract malformations. These ubiquitous plasticizers, found in myriad commercial, medical, and cosmetic products, readily leach from flexible polyvinyl chloride (PVC) plastics (Thomas and Thomas 1984), leading to widespread human exposure (Blount et al. 2000). Of particular concern as a vulnerable population are critically ill neonates, who may receive up to 20 mg/day of phthalate esters (Loff et al. 2000) through treatment using phthalate-containing medical devices. Accordingly, developmental exposure to phthalate esters has prompted an expression of “serious concern” by an Expert Panel of the Center for the Evaluation of Risks to Human Reproduction (Kavlock et al. 2006). To date, however, the response of the male human reproductive system to developmental phthalate exposure remains unknown.

Male rats exposed *in utero* to di(*n*-butyl) phthalate (DBP) exhibit numerous reproductive tract defects, including decreased anogenital distance (AGD), formation of multinucleated germ cells (MNG), hypospermatogenesis, underdeveloped or absent reproductive organs, cryptorchidism, and hypospadias (Gray et al. 2000; Mylchreest et al. 1998, 2000). Many of these effects result from insufficient testosterone production by the fetal testis during a critical fetal masculinization window (Welsh et al. 2008), due to rapid (within hours) suppression of genes encoding cholesterol transport/biosynthetic and steroidogenic proteins in fetal Leydig cells (Lehmann et al. 2004; Thompson et al. 2004). Interestingly, when the rat model of *in utero* phthalate exposure was extended to the mouse, no suppression of testicular testosterone or its biosynthetic genes was observed (Gaido et al. 2007; Johnson et al. 2011). However, both species exhibited enlarged seminiferous cords and MNG formation. The disparity in response suggests a species-specific sensitivity to phthalate-induced suppression of androgen biosynthesis, and invites speculation as to the human response. Importantly, *in vitro* approaches using mouse Leydig cells and fetal rat and human testes have thus far shown inconsistent responses in both steroidogenic and germ cell end points (Chauvigne et al. 2009; Clewell et al. 2010; Hallmark et al. 2007; Lambrot et al. 2009; Lehraiki et al. 2009) compared with the *in utero* response. These technical limitations suggest cultured fetal testes and testicular cells to be a poor phthalate endocrine-disruption model. In this article, we describe the development of a human fetal testis xenotransplant bioassay to directly assess the human response to phthalate exposure.

## Materials and Methods

*Animals.* Adult male Crl:NIH-Foxn1^rnu^ nude rats (strain code 316) and BALB/c nude mice (strain code 194) 8–10 weeks of age were obtained from Charles River Laboratories (Wilmington, MA) and used as hosts for xenotransplant surgeries. Pregnant time-mated Fischer rats (strain code 002), C57BL/6NCrl mice (strain code 027), and CD-1 mice (strain code 022) were purchased from Charles River Laboratories (plug day is considered day 0). All animals were maintained in a temperature- and humidity-controlled vivarium with a 12-hr alternating light–dark cycle. Animals were housed in community cages with free access to water and Purina Rodent Chow 5001 (Farmer’s Exchange, Framingham, MA). The Brown University Institutional Animal Care and Use Committee approved all experimental protocols in compliance with the National Institutes of Health guidelines (National Research Council 1996). All animals were treated humanely and with regard for alleviation of suffering.

*Human testis.* Human fetal testes were obtained under sterile conditions from spontaneously aborted fetuses (gestational weeks 10–24) at Women and Infants’ Hospital (Providence, RI). All experiments complied with all applicable U.S. requirements and were conducted in accordance with the Institutional Review Board protocol “A Xenotransplant Bioassay To Predict Testicular Dysgenesis Syndrome” (Project No. 07-0093; Women and Infants’ Hospital). Full informed written parental consent was obtained in compliance with institutional guidelines. Testes were washed in Hank’s Balanced Salt Solution without magnesium chloride, calcium chloride, magnesium sulfate, or phenol red, supplemented with gentamicin (50 μg/mL) and penicillin/streptomycin (50 μg/mL) (Gibco, Grand Island, NY). Testes were then transferred to ice-cold transport media consisting of Leibovitz’s L-15 medium (Invitrogen, Grand Island, NY) supplemented with gentamicin (50 μg/mL) and penicillin/streptomycin (50 μg/mL) for transport to Brown University.

*Surgical procedures.* Rat and mouse source testes were obtained by pup dissection following dam sacrifice with an overdose of isoflurane on gestational day (GD) 15 (mice) or GD16 (rats). Left and right gonads were visualized using a Nikon SMZ-U dissecting microscope (Nikon Instruments, Melville, NY), separated from adjacent mesonephros, and transferred to individual labeled wells of ice-cold transport media until xenografting. Human fetal testes were sectioned with a sterile scalpel, cut into approximately 1-mm^3^ fragments, and held in ice-cold transport media until xenografting. For surgery, hosts were anesthetized with isoflurane (Baxter Healthcare Corporation, Deerfield, IL), and the abdomen was shaved and scrubbed with 10% povidone–iodine solution (Purdue Products LP, Stamford, CT). A 4-cm abdominal incision was made to exteriorize each kidney, and a 2-mm opening was made in the renal capsule. For rat and mouse xenografts, six to eight left testes from individual pups were xenografted into control hosts between the left and right kidneys. The corresponding right testes were xenografted into DBP-treated hosts in a similar manner. Implant locations were diagrammatically recorded such that left and right testes from individual pups could be matched upon collection to allow for direct comparisons. For human xenografts, each human specimen served as its own control, with control and DBP-treated hosts each xenografted with six to eight fragments from an individual fetal human donor into control hosts between the left and right kidneys. The peritoneum was closed with 5-0 Vicryl sutures (Ethicon, Inc., Somerville, NJ) and the incision was stapled closed with stainless steel wound clips (Becton Dickinson, Sparks, MD). Selected animals were implanted with a single Alzet osmotic minipump (model #2001; Durect, Cupertino, CA) containing a solution of 0.3 M bromodeoxycytidine (BrdC; Sigma-Aldrich, St. Louis, MO). BrdC is converted to bromodeoxyuridine (BrdU) in cells and detected by immunohistochemical labeling. Minipumps were implanted subcutaneously at the dorsal midthoracic midline immediately after testis xenografting. Incisions were closed using wound clips. Following surgery, animals were monitored daily for food and water intake and any obvious signs of stress.

*Phthalate exposure.* DBP and corn oil were purchased from Sigma-Aldrich. At 24 hr after surgery, hosts were given a single dose of 100, 250, or 500 mg/kg DBP (100DBP, 250DBP, and 500DBP, respectively) in corn oil vehicle (1 mL/kg) or corn oil vehicle alone (control) by oral gavage. Some hosts were given additional doses 24 and 48 hr after initial treatment for a total of 1, 2, or 3 consecutive days of treatment. Six hours after the final dose, hosts were euthanized by an overdose of isoflurane and kidneys were removed. For AGD assessment, pregnant female CD-1 mice were treated with 500DBP in corn oil vehicle (1 mL/kg) or vehicle alone (control) once per day from GD14 to GD18.

*AGD assessment.* At postnatal day (PND) 3 (the day of birth is PND1), treatment-blinded pups exposed to either DBP or corn oil vehicle (control) *in utero* were weighed, and AGD (distance from the base of the phallus to the anal orifice) was measured using a stereomicroscope fitted with a 1-cm reticule (0.1-mm increments).

*Fetal rat testis culture.* A single GD17 rat testis was cultured at the air/liquid interface on 0.4 µm Millicell-CM filters (Millipore, Billerica, MA) in a six-well polystyrene culture dish (Corning Inc., Corning, NY) with 1.1 mL of medium for 24 hr at 37°C. Basal medium was DMEM/F12 (Invitrogen) containing 1X insulin, transferrin, selenium supplement (Sigma-Aldrich), 1 mM sodium pyruvate (Sigma-Aldrich), and 25 mM HEPES, pH 7.4 (Sigma-Aldrich) (adapted from Hallmark et al. 2007). After placing the testis on the filter, 25 μL of medium was placed on the testis. For testes exposed to human chorionic gonadotropin (Sigma-Aldrich), we used a final concentration of 0.1 IU. For testes exposed to monobutyl phthalate (MBP; Sigma-Aldrich), 250 µM MBP was included in the medium. This MBP concentration approximates the maximum levels observed in fetal rat serum after maternal gavage of 250DBP (Clewell et al. 2008). After culturing, we purified testis total RNA and quantified gene expression relative to *Tbp* (TATA box binding protein).

*Quantitative reverse-transcription polymerase chain reaction (qRT-PCR).* Testes or testis fragments were dissected from the capsule and flash frozen in liquid nitrogen for qRT-PCR analysis. Total RNA was isolated using STAT-60 reagent (Tel-Test Inc., Friendswood, TX) and chloroform:isoamyl alcohol elution in a phase-lock gel tube. Cleanup of isolated RNA was performed using a RNeasy Mini kit (QIAGEN, Valencia, CA) following the manufacturer’s protocol. After isolation and cleanup, samples were eluted in 50 μL of RNase free water (Invitrogen) and RNA concentration was determined using a NanoDrop spectrophotometer (Thermo Scientific, Wilmington, DE). RNA was transcribed to cDNA using the iScript™ cDNA Synthesis Kit (Bio-Rad, Hercules, CA) for human xenografts, or the High Capacity cDNA Reverse Transcription kit (Applied Biosystems, Foster City, CA) for rat and mouse xenografts. qRT-PCR was performed on a Prism 7900HT Sequence Detection System (Applied Biosystems) using Taqman® Gene Expression assays: *Cyp11a1* (cytochrome P450, family 11, subfamily A, polypeptide 1), *Cyp17a1* (cytochrome P450, family 17, subfamily A, polypeptide 1), *Scarb1* (scavenger receptor class B, member 1), *Star* (steroidogenic acute regulatory protein), *Insl3* [insulin-like 3 (Leydig cell)], and *Gapdh* (glyceraldehyde-3-phosphate dehydrogenase) for xenotransplants; and *Cyp11a1, Cyp17a1, Tbp, Gnrhr* (gonadotropin-releasing hormone receptor), and *Smpx* (small muscle protein, X-linked) for *in vitro* rat testis cultures (Applied Biosystems) according to the manufacturer’s protocol [see Supplemental Material, Table S1 (http://dx.doi.org/10.1289/ehp.1104711)]. Cycle threshold values were determined using SDS 2.3 software (Applied Biosystems), and target gene expression was calculated relative to *Gapdh* (xenografts) or *Tbp* (*in vitro* rat testis culture) using Relative Quantification Manager Software (Applied Biosystems).

*Histopathology.* Selected implants were left in the kidney and fixed for 24 hr in modified Davidson’s solution, postfixed in 10% neutral buffered formalin, and stored at 4°C before embedding. Sagittal slices of kidney containing implants were dehydrated in graded ethanol baths, embedded in glycol methacrylate (Technovit 7100; Heraeus Kulzer GmBH, Wehrheim, Germany), sectioned (5 μm), stained with hematoxylin and eosin (H&E), and scanned into an Aperio ScanScope CS microscope (Aperio Technologies, Vista, CA). Treatment-blinded slides were quantified for total seminiferous cord area (counting all seminiferous cords with at least one identifiable germ cell) using ImageScope software (Aperio), and then quantified for the total number of mononuclear and multinuclear germ cells within defined seminiferous cords using an Olympus BH-2 microscope (Olympus, Center Valley, PA). Germ cells were identified based on location, nuclear size, and eosinophilic staining compared with Sertoli cells. MNGs were defined as two or more identifiable nuclei sharing the same cytoplasm.

*Immunohistochemistry.* Sagittal slices of kidney containing implants were processed in paraffin, sectioned, and processed for horseradish avidin-biotin immunoperoxidase staining (SP-2001; Vector Laboratories, Burlingame, CA) using a mouse monoclonal CD31 anti-human antibody (Dako Cytomation, Carpinteria, CA). Antibody binding was revealed using 3,3´-diaminobenzidine, and slides were counterstained with hematoxylin. For BrdU staining, glycol methacrylate sections were prepared and stained using a primary monoclonal mouse anti-BrdU antibody (Dako Cytomation) as described previously (Allard et al. 1995). Sections were counterstained with periodic acid–Schiff/hematoxylin.

Ex vivo *testosterone production.* At harvest, three Fischer rat (GD18) or C57BL6 mouse (GD17) testis implants were immediately transferred into individual wells of a cell culture plate containing 500 µL of custom M-199 media (Invitrogen) containing no phenol red and reduced methionine. Implants were incubated on a rocker at 37°C for 3 hr, and media was collected into sterile, low-adhesion microcentrifuge tubes (USA Scientific, Ocala, FL) and frozen at –80°C until analysis. Testosterone was quantified by a commercial radioimmunoassay kit (limit of detection, 7.2–895.9 ng/dL; coefficient of variation interassay, 7.0%; intraassay, 1.7%) at The University of Virginia Center for Reproduction in Research Ligand Assay and Analysis Core (Charlottesville, VA; supported by the *Eunice Kennedy Shriver* National Institute of Child Health and Human Development, Specialized Cooperative Centers Program in Reproduction and Infertility Research, National Institutes of Health (grant U54-HD28934).

## Results

In utero *DBP exposure does not alter mouse AGD.* AGD, a postnatal marker of testosterone action during the fetal male masculinization window, is reduced by *in utero* phthalate exposure in the rat (Macleod et al. 2010). To determine if *in utero* DBP exposure in mice reduces AGD, we measured AGD on PND3 in animals exposed to 500DBP from GD14 to GD18. Unlike in rats, fetal 500DBP exposure in mice did not reduce AGD [see Supplemental Material, Figure S1 (http://dx.doi.org/10.1289/ehp.1104711)].

**Figure 1:**
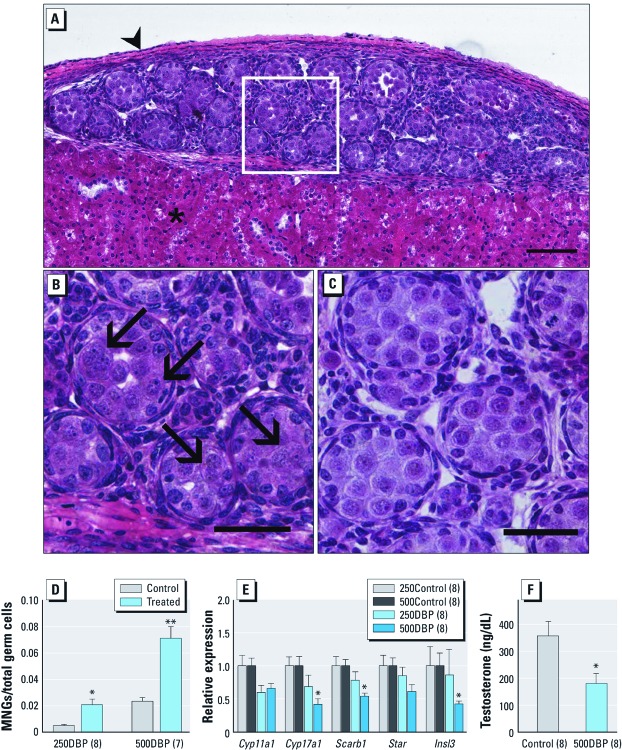
Photomicrographs (H&E) and quantitation of effects of DBP on seminiferous cords and Leydig cells of GD16 Fischer rat testis implanted into adult male immunodeficient host rats, which were then exposed for 2 days to 250DBP, 500DBP, or corn oil vehicle (control). (*A*) Low power (bar = 100 μm) and (*B*) high power (bar = 50 μm) view after 500DBP treatment showing the kidney capsule (arrowhead; *A*), the renal parenchyma (asterisk; *A*), and MNGs (arrows; *B*). (*C*) Control at high power (bar = 50 μm). (*D*) Quantification of total MNGs. (*E*) Steroidogenic gene expression relative to *Gapdh.* (*F*) *Ex vivo* testicular testosterone production. In *D–F*, values are mean ± SE; values shown in parentheses are the number of hosts. *p < 0.05, and **p < 0.01 compared with the corresponding control by two-tailed paired *t*-test. For 500DBP treatment in *E*, *p* = 0.09 for*Cyp11a1* and *p* = 0.06 for *Star*.

*Rodent xenografts recapitulate* in utero *phthalate exposure phenotypes.* Before testing the human response to phthalates, we needed to determine that the transplant model recapitulated both the *in vivo* Leydig cell and seminiferous cord effects of phthalates observed in rats and mice. Therefore, we harvested fetal testes from GD16 Fischer rat pups and immediately grafted them into the renal subcapsular space of adult male immunodeficient rat hosts. Following grafting, hosts were dosed once daily for 2 days with 250DBP or 500DBP or corn oil vehicle (control) by oral gavage. Implants were harvested 6 hr after the final dose and processed for histopathology, gene expression, or testosterone production end points. Xenografts appeared viable, containing intact seminiferous cords surrounded by interstitial spaces with occasional blood vessels, proliferating cells, and minimal apoptosis (Figure 1A). Treatment with either 250DBP or 500DBP induced the formation of MNGs (Figure 1B–D), with no treatment-related effect on total germ cell numbers [see Supplemental Material, Figure S2 (http://dx.doi.org/10.1289/ehp.1104711)]. qRT-PCR analysis revealed a dose-dependent decrease in expression of the Leydig cell–specific gene involved with testicular descent, *Insl3*, and the steroidogenic genes *Cyp17a1 and Scarb1* (Figure 1E). Reduction in expression approached significance for the remaining steroidogenic genes (*Cyp11a1* and *Star*; *p* < 0.09). In addition, we observed a significant decrease in testosterone production in implants exposed to 500DBP (Figure 1F). Together, these results paralleled both the seminiferous cord (MNG formation) and Leydig cell (suppressed steroidogenesis) end points observed in fetal rats following gestational DBP exposure, providing a proof-of-principle for our transplant bioassay.

**Figure 2:**
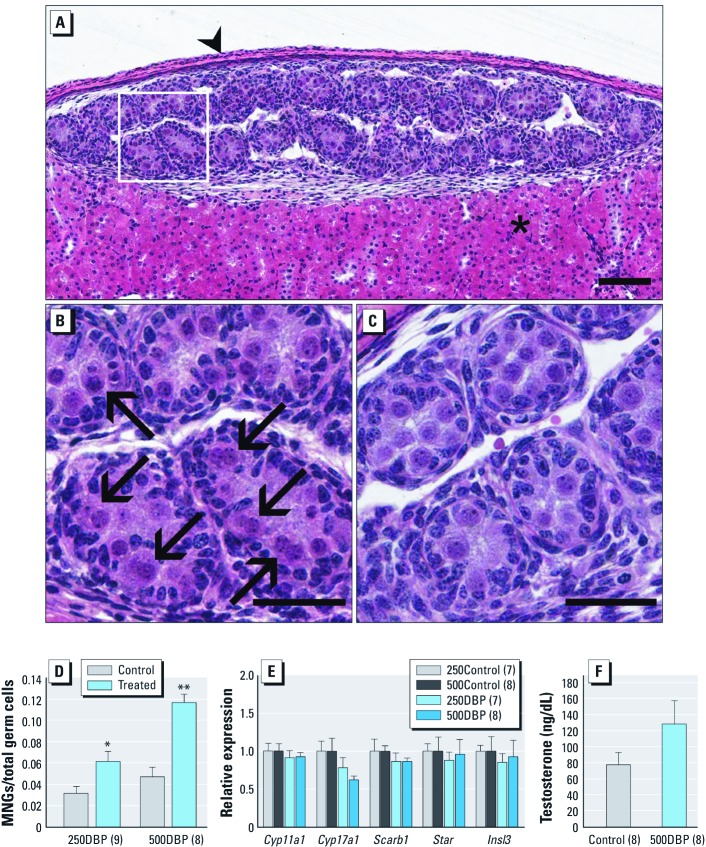
Photomicrographs (H&E) and quantitation of effects of DBP on seminiferous cords and Leydig cells of GD15 C57BL6 mouse testis implanted into adult male immunodeficient host rats, which were then exposed for 2 days to 250DBP, 500DBP, or corn oil vehicle (control). (*A*) Low power (bar = 100 μm) and (*B*) high power (bar = 50 μm) view after treatment with 500DBP showing the kidney capsule (arrowhead; *A*), renal parenchyma (asterisk; *A*), and MNGs (arrows; *B*). (*C*) Control at high power (bar = 50 μm). (*D*) Quantification of total MNGs. (*E*) Steroidogenic gene expression, relative to *Gapdh*. (*F*) *Ex vivo* testicular testosterone production. In *D–F*, values are mean ± SE; numbers in parentheses are the number of hosts. *p < 0.05, and **p < 0.01 compared with the corresponding control by two-tailed paired *t*-test. For *Cyp17a1* in *E*, *p* = 0.08 for 250DBP and *p* = 0.08 for 500DBP.

To determine whether the *in utero*–consistent effects were maintained with mice, we xenografted GD15 C57BL6 mouse testes into adult immunodeficient rat hosts and exposed them to DBP using the same transplant and exposure paradigm. Xenografts exposed to 250DBP or 500DBP exhibited the same significant induction of MNGs observed in rats (Figure 2A–D). However, we observed no overall decrease in gene expression at either dose (Figure 2E) and only a slight increase in testosterone production (Figure 2F), similar to the response in the intact fetal mouse (Gaido et al. 2007).

In these initial experiments, rat or mouse testes were grafted into the “permissive” rat host—a species that exhibits effects on both germ cells and Leydig cells after phthalate treatment. To rule out the responses as dependent on host species, we grafted either mouse or rat fetal testes into immunodeficient mouse hosts and exposed animals to DBP in a similar manner. Our results confirmed MNG formation in both mouse and rat testis grafts. Expression of *Cyp17a1* was reduced significantly in fetal mouse testis grafts [see Supplemental Material, Figure S3 (http://dx.doi.org/10.1289/ehp.1104711)]. No overall suppression was observed in the remaining genes. The *in utero*–consistent effects in these two host species demonstrated that the host species does not determine the grafted testis response to DBP and that the response to DBP is intrinsic to the testis itself, thereby allowing for the testing of the human fetal testis response to phthalate exposure.

**Figure 3:**
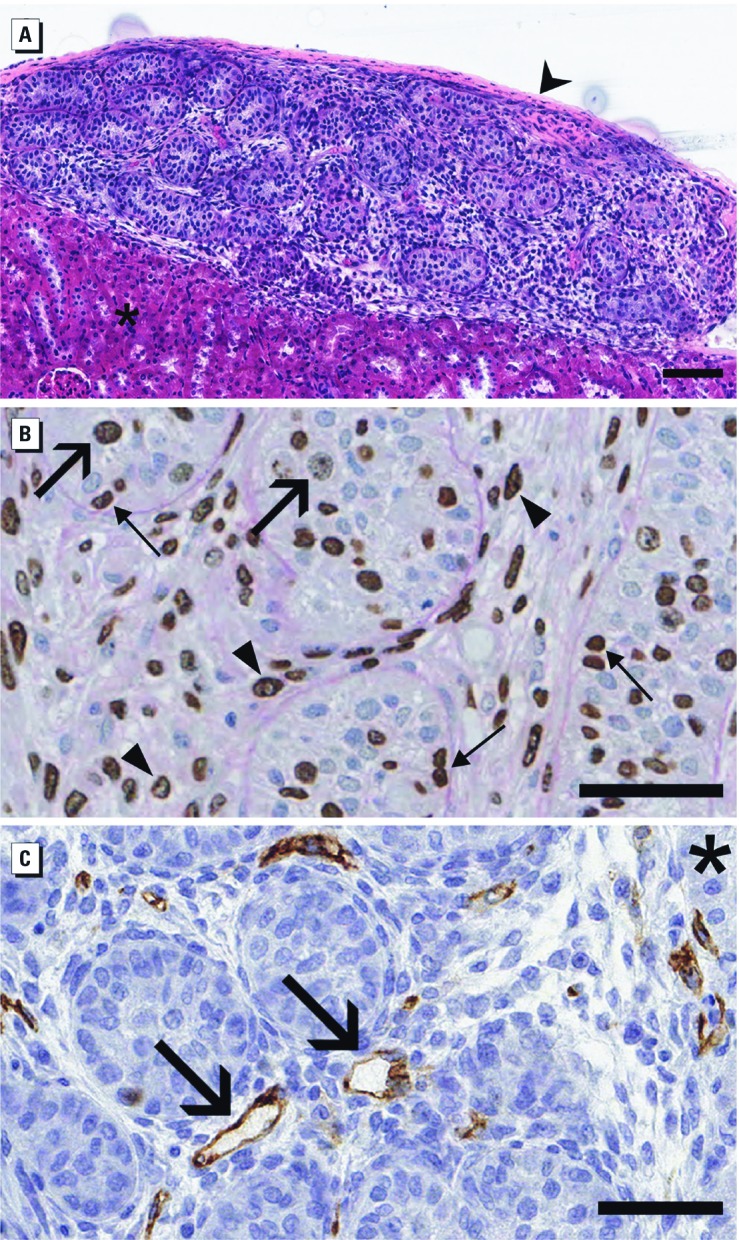
Photomicrographs of control human fetal testis implanted into adult male immunodeficient host rats. (*A*) Low-power view of 22-week-old fetal testis after 2 days (H&E) showing the kidney capsule and the renal parenchyma (asterisk); bar = 100 μm. (*B*) BrdU labeling of 19-week-old fetal testis after 3 days (periodic acid–Schiff hematoxylin counterstain) showing germ cells (large arrows), Sertoli cells (thin arrows), and interstitial cells (arrowheads); bar = 50 μm. (*C*) Human anti-CD31 staining of 22-week-old fetal testis after 3 days (hematoxylin counterstain) showing blood vessels with lumen (arrows) and kidney parenchyma (asterisk); bar = 50 μm.

*Phenotype of phthalate-exposed human fetal testis xenografts.* Human fetal testes were obtained under sterile conditions from spontaneously aborted fetuses. All human specimens were histologically normal, with an average gestational age of 18.6 weeks, and average postmortem interval of < 23 hr between delivery and xenografting (Table 1). With each testis serving as its own control, testes were diced into fragments approximately 1 mm^3^ in size, xenografted into immunodeficient adult rat hosts, and exposed to DBP or vehicle. Two days after grafting, testis xenografts were viable, with obvious cellular mitosis and normal testicular architecture (Figure 3A). BrdU labeling demonstrated active mitosis of germ cell, Sertoli cell, and interstitial cell populations (Figure 3B). Xenografts also contained identifiable blood vessels stained with human anti-CD31 (Figure 3C) both within the xenograft and at the interface with the kidney parenchyma.

**Table 1:** Human fetal testis used as source tissue in xenografts.

Donor	Gestational age (weeks)	PMI (hr)	Relevant pathology notes
1		10		17.5		
2		13.4		6.0		
3		14		8.0		
4		16.5		25.5		Multiple fetal anomalies
5		17		30.0		
6		17a		34.5		Chorioamnionitis
7		17a		34.3		Chorioamnionitis
8		18		34.0		
9		18		13.5		Anencephaly
10		18a		25.0		
11		18a		25.0		
12		18		23.0		Premature membrane rupture
13		19		14.5		Abdominal wall defect
14		19		25.0		Polycystic kidneys
15		19		14.5		Multiple fetal anomalies
16		19		<12		
17		19.3		23.0		Neural tube defect
18		19.57		24.0		Trisomy 13/18
19		20		26.0		Diaphragmatic hernia
20		21a		35.0		
21		21a		35.0		
22		22		12.0		Premature membrane rupture
23		22		23.0		
24		22		26.0		Premature membrane rupture
25		22		24.0		Premature membrane rupture
26		23		25.0		Preterm labor
PMI, postmortem interval (number of hours between fetal delivery and xenografting of testis tissue). aTwins.						

**Figure 4:**
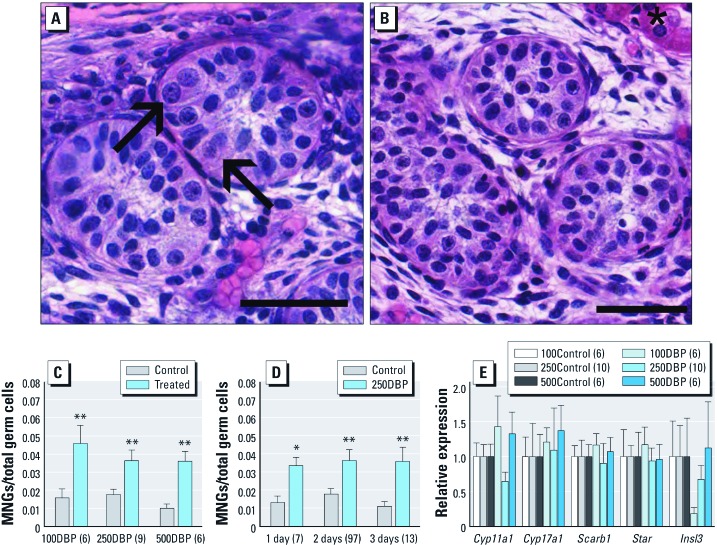
Photomicrographs (H&E) and quantitation of human fetal testis implanted into adult male immunodeficient rat hosts, which were than exposed to 100DBP, 250DBP, 500DBP, or corn oil vehicle (control). High power view of human fetal testis treated for 2 days with 500DBP (*A*) or corn oil vehicle (*B*) showing MNGs (arrows) and kidney parenchyma (asterisk); bar = 50μm. Quantification of total MNGs by dose (*C*) and time (*D*). (*E*) Steroidogenic gene expression after 2 days, relative to *Gapdh.* Significant differences were observed among treatment groups for *Cyp17a1* and *Insl3* only (Bartlett’s test); therefore, data were analyzed by Wilcoxon matched-pairs signed rank test, which found no significant differences at any dose for either *Cyp17a1* or *Insl3* (all *p *> 0.15). Pairwise comparisons between treatment groups and control for the remaining genes were analyzed by paired *t*-test. In *C–E*, values are mean ± SE. Values in parentheses are the number of human specimens. *p < 0.05, and **p < 0.01 compared with the corresponding control by two-tailed paired *t*-test.

Exposure of human xenografts to 100DBP, 250DBP, or 500DBP caused a significant increase in MNG content (Figure 4A,C) compared with control (Figure 4B,C), demonstrating this *in vivo* histopathological hallmark of phthalate exposure in humans for the first time. MNG induction was rapid (within 6 hr of exposure), with a consistency of effect across various doses and multiple days (Figure 4C,D). Further, we observed no treatment-related effect on germ cell content across a range of doses or days [see Supplemental Material, Figure S2E,F (http://dx.doi.org/10.1289/ehp.1104711)]. Control xenografts exhibited no significant change in steroidogenic gene expression across 1, 2, or 3 days, indicating no time dependent decrease in steroidogenesis (see Supplemental Material, Figure S4). A range of DBP doses did not significantly alter expression of any Leydig cell steroidogenic genes, relative to control, as determined by paired *t*-test (Figure 4E). Although variance between controls and DBP doses differed significantly for *Cyp17a1* and *Insl3* (*p* < 0.05), no significant differences were observed between control and any DBP dose for either gene by Wilcoxon matched-pairs signed rank test (Figure 4E). Although the shape of the *Insl3* dose response is suggestive of low dose sensitivity, the difference was not significant. The apparent trend may be the result of random variation or a magnitude of change too small to detect given the current sample size. Together, these data demonstrate that humans, mice, and rats are sensitive to MNG formation across a range of doses of DBP, including low doses within one order of magnitude of potential preterm neonatal exposures.

## Discussion

The species-specific sensitivity to developmental phthalate exposure highlights an emerging problem in the assessment of chemical safety: relying on model-species approaches to determine mode of action and pathway responses to toxicant exposure. As exemplified by *in utero* exposure to phthalates in mice and rats, species differences can be fundamentally important in determining susceptibility to toxicant exposure. Indeed, the use of non-rodent and rodent species for predicting target organ toxicity exhibits only a 70% true positive concordance rate (Olson et al. 2000), indicating that a mismatch between animal and human responses is still common. To address this issue, we directly assessed the human response to phthalate exposure using a cross-species xenotransplantation approach, which has previously been explored as a model of testicular development and function in rodents (Kuopio et al. 1989), primates (Ehmcke et al. 2011), and humans (Mitchell et al. 2010). The *in utero*–consistent responses we observed with immunodeficient rat and mouse hosts demonstrate that the response is intrinsic to the testis itself and host independent, providing a suitable model for examining the human response without influence from host-derived factors.

Liu et al. (2008) reported antiandrogenic effects, such as hypospadias and reduced AGD, in mice exposed gestationally to phthalates. However, in the AGD analysis the pup rather than the dam was used as the statistical unit, which contrasts with standard methods used by others (Barlow and Foster 2003; Mylchreest et al. 1998). In a reanalysis of mouse AGD data from Liu et al. (2008), using the means and SDs reported in their Table 1 but with the litter (*n* = 10) as the statistical unit, we observed no significant change in AGD at any DBP dose level; *p*-values from analysis of variance with Dunnett’s post hoc test were all > 0.36. Further, decreased *Insl3* and testosterone have been observed in certain Chinese strains of mice, which have not been studied elsewhere (Wu et al. 2010). *In vitro* studies of whole testis or Leydig tumor cell lines have also suggested that the mouse is sensitive; however, the responses have been widely varied, with reports of increases or decreases in steroidogenesis with phthalate treatment (Dees et al. 2001; Fan et al. 2010; Gunnarsson et al. 2008; Wang et al. 2007), which can be influenced by factors such as age, culture duration, or use of exogenous hormones (Lehraiki et al. 2009).

Both epidemiological and *in vitro* approaches have been used previously to examine the human response to phthalates. Although epidemiological approaches directly address the human response, they are limited by their associative nature. It is difficult to establish causal relationships between early life exposures and later-life health outcomes based solely on observational studies. Therefore, mechanistic research is needed to establish a basis for a hypothetical fetal origin of an adult disease. *In vitro* organ culture allows for directly testing the target species in a controlled-exposure environment. However, several contradictory effects highlight the limitations of *in vitro* approaches compared with the *in vivo* response, for example, treatment-related germ cell apoptosis (Boekelheide et al. 2009; Chauvigne et al. 2009; Lambrot et al. 2009), steroidogenic and nonsteroidogenic gene expression [see Supplemental Material, Figure S5 (http://dx.doi.org/10.1289/ehp.1104711)], and testosterone production (Hallmark et al. 2007; Stroheker et al. 2006), as well as a requirement for stimulation of the luteinizing hormone receptor with *in vitro* cultures that is not required to maintain fetal testis steroidogenesis *in utero* (Hallmark et al. 2007). In addition, MNG formation, an important conserved phthalate-induced seminiferous cord effect observed across all species examined thus far *in vivo*, has not been reported with *in vitro* fetal testis culture to date. These technical and treatment-related challenges suggest that *in vitro* conditions are inadequate for functional assessment of at least some toxicant-induced fetal testicular responses to phthalates, providing a rationale for the development of the complex xenotransplant system described here. Indeed, while we were performing our xenotransplant experiments, Mitchell et al. (2012) reported a similar approach of xenografting human fetal testes to examine the response to phthalates. Although these models differ in their exposure duration and host–environment parameters, Mitchell et al. also observed no suppression of steroidogenesis with phthalate treatment, corroborating the results presented here.

Two aspects of this xenotransplant model deserve further discussion, namely, the phthalate dose to the graft and the testosterone assay. The induction of MNG formation across a range of doses (Figure 4C) and exposure time (Figure 4D) demonstrates that sufficient toxicant is delivered to the graft to produce this consistent effect. In studies using pregnant rats, the dose level required to induce MNG formation and to decrease steroidogenesis in the fetal testis is similar at 50–100 mg/kg/day DBP (Boekelheide et al. 2009; Hallmark et al. 2007; Lehmann et al. 2004). The results from the testosterone assay performed with the human fetal testes were highly variable. For testosterone assays of rat and mouse fetal testis, the entire fetal gonad was removed from the transplant site and placed in short-term culture, allowing consistency across samples, all of which were the same gestational age. The human xenografts consisted of variably sized fragments from different parts of the fetal testis across a range of gestational ages, including the period just beyond the masculinization window (up to 14 weeks). Still, there is a high correlation between steroidogenic gene expression and testicular testosterone (Thompson et al. 2005), and we observed no time-dependent change in gene expression in controls. Therefore, this study and other studies of phthalate exposure of the fetal testis suggest that steroidogenic gene expression can act as an appropriate surrogate measure of testosterone production.

In the present study, we observed a rapid and robust induction of MNG formation across a range of doses in all three species examined. Phthalate-induced effects on the seminiferous cords include delayed germ cell development (Ferrara et al. 2006), and MNGs have been suggested to result from altered support and signaling from fetal Sertoli cells after developmental DBP exposure (Kleymenova et al. 2005). MNGs are typically eliminated by PND10 in the mouse (Saffarini et al. 2011) and PND16 in the rat (Barlow and Foster 2003), but abnormal mitosis of MNGs has been observed in the early postnatal period in rats exposed to DBP *in utero* (Kleymenova et al. 2005). Multinucleated spermatogonia have been observed in the testes of both juvenile cryptorchid boys (Cortes et al. 2003) and adult men (Nistal et al. 2006) presenting with carcinoma *in situ* of the testis. Although MNGs do not express the primordial germ cell/gonocyte markers observed in carcinoma *in situ* cells (Saffarini et al. 2011), the long-term effects of these dysgenetic germ cells remains unclear. For these reasons, phthalate exposure and the effects on seminiferous cords we observed in this study, which were manifested as MNG formation, raise some concern and identify the need for further evaluation of these end points.

## Conclusions

The TDS hypothesis suggests that increases in male reproductive tract abnormalities may result from developmental exposure to EDCs such as DBP. Although the fetal rat is sensitive to the antiandrogenic effects of DBP, human fetal testis xenografts exposed to DBP showed no decrease in expression of the steroidogenic genes responsible for fetal testosterone biosynthesis. However, our observation of phthalate-induced effects on seminiferous cords, manifested as MNG induction, warrants further exploration.

## Supplemental Material

(1.2 MB) PDF
