# Supplementary material for: Human Fetal Testis Xenografts Are Resistant to Phthalate-Induced Endocrine Disruption
Source: Environ Health Perspect. 2012 Apr 17;120(8):1137–43. doi: 10.1289/ehp.1104711 (PMC3440087; doi:10.1289/ehp.1104711)
Supplement: (1.2 MB) PDF [file ehp.1104711.s001.pdf]

Supplemental Material

Human Fetal Testis Xenografts Are Resistant To Phthalate-Induced Endocrine Disruption

Nicholas E. Heger, Susan J. Hall, Moses A. Sandrof, Elizabeth V. McDonnell, Janan B. Hensley, Erin N. McDowell, Kayla A. Martin, Kevin W. Gaido, Kamin J. Johnson, and Kim Boekelheide

Table of Contents

Table S1

Figure S1

Figure S2

Figure S3

Figure S4

Figure S5

Supplemental Material, Table S1

Gene	Assay Number<br>Rat	 Mouse	 Human
CYP11A1	Rn00568733_m1	Mm00490735_m1	Hs00167984_m1
CYP17A1	Rn00562601_m1	Mm00484040_m1	Hs00164375_m1
SCARB1	Rn00580588_m1	Mm00450236_m1	Hs00194092_m1
STAR	Rn00580695_m1	Mm00441558_m1	Hs00264912_m1
INSL3	Rn00586632_m1	Mm01340353_m1	Hs01895076_s1
GAPDH	Rn99999916_s1	Mm99999915_g1	Hs99999905_m1
SMPX	Rn00584554_m1		
GHRHR	Rn00578981_m1		
TBP	Rn01455646_m1		

Supplemental Material, Table S1. Taqman® Gene Expression assays for qRT-PCR analysis of rat, mouse, and human tissue.

Supplemental Material, Figure S1

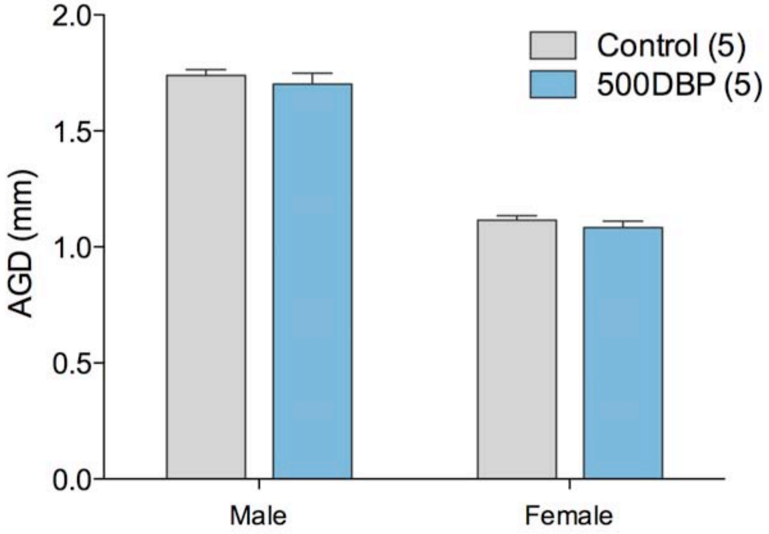

Supplemental Material, Figure S1. AGD of PND 3 CD1 male and female mice following daily gestational exposure to 500DBP or control from gd 14-18. All values are mean ± SEM. N=litters/group (values listed in parentheses). The AGD in all animals of a litter was averaged to determine litter mean. No differences were observed when corrected for pup body weight (data not shown).

Supplemental Material, Figure S2

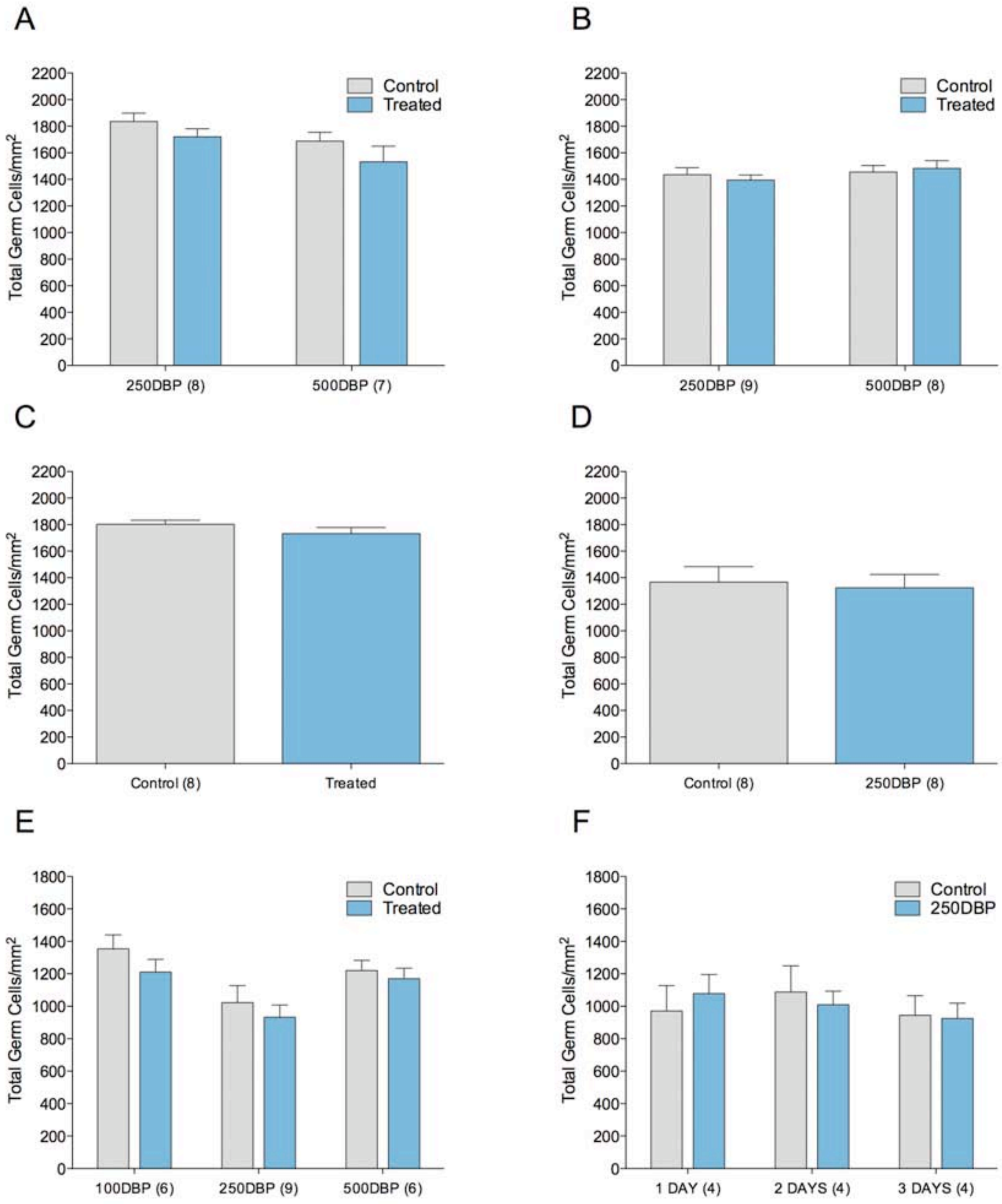

Supplemental Material, Figure S2. Effect of DBP treatment on total germ cell numbers. Total number of germ cells/mm^2 in rat-into-rat (A), mouse-into-rat (B), rat-into-mouse (C), or mouse-into-mouse (D) xenografts following 2 days treatment with 250 or 500DBP. (E-F) Total number of germ cells/mm^2 in human-into-rat xenografts following

treatment with 100DBP, 250DBP, or 500DBP for 1, 2 or 3 days. All values are mean ± SEM. n=host (rat and mouse xenografts), or specimen (human xenografts); values listed in parentheses. Germ cells were counted as described in Materials and Methods. Data analyzed by paired *t*-test vs. corresponding control; no significant differences observed.

Supplemental Material, Figure S3

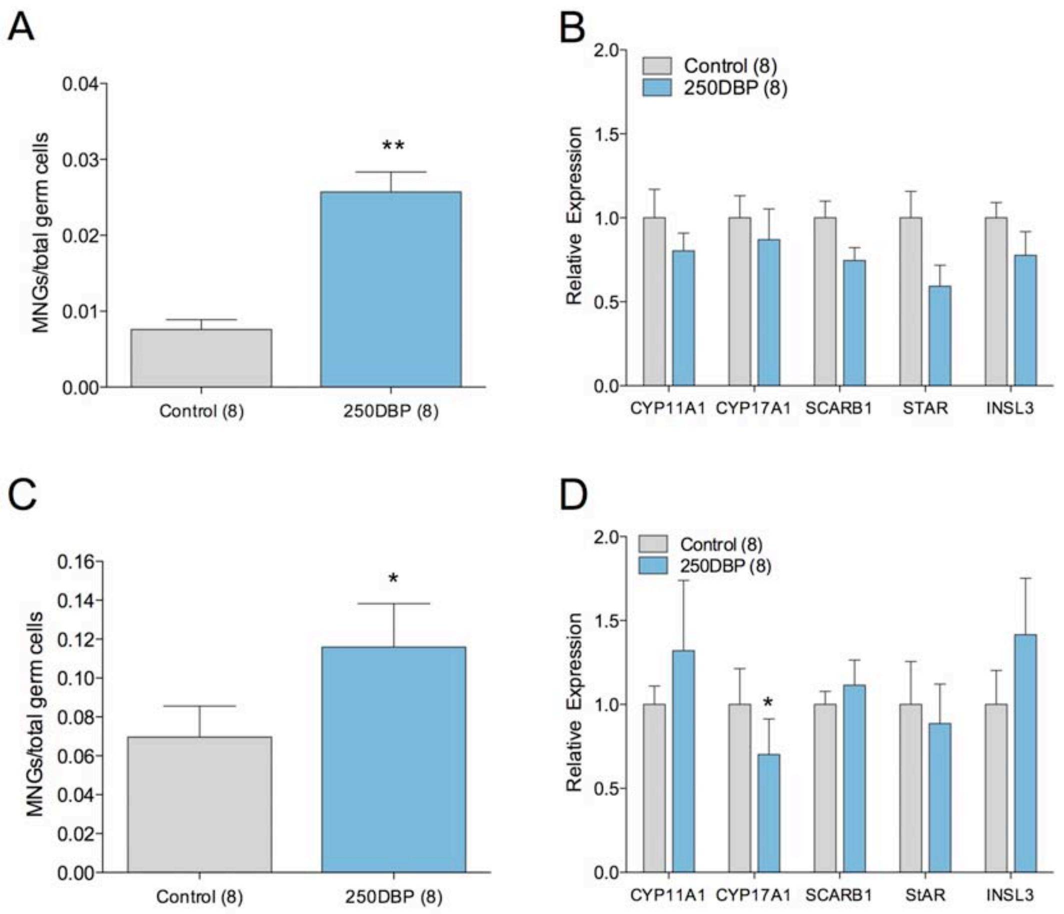

Supplemental Material, Figure S3. Seminiferous cord and Leydig cell effects of gd 16 Fischer rat or gd 15 C57BL6 mouse testis implanted into adult male immunodeficient mouse hosts and dosed for 2 days with 250mg/kg DBP or control. Quantification of MNGs/total germ cells (A) and steroidogenic gene expression (B) in rat-into-mouse xenografts. Quantification of MNGs/total germ cells (C) and steroidogenic gene expression (D) in mouse-into-mouse xenografts. All values are mean ± SEM; n=host (values listed in parentheses). * $p<0.05$; ** $p<0.01$ by two-tailed paired *t*-test vs. corresponding control. (B) *Scarb1* $p = 0.08$ vs. control; *Star* $p = 0.06$ vs. control.

Supplemental Material, Figure S4

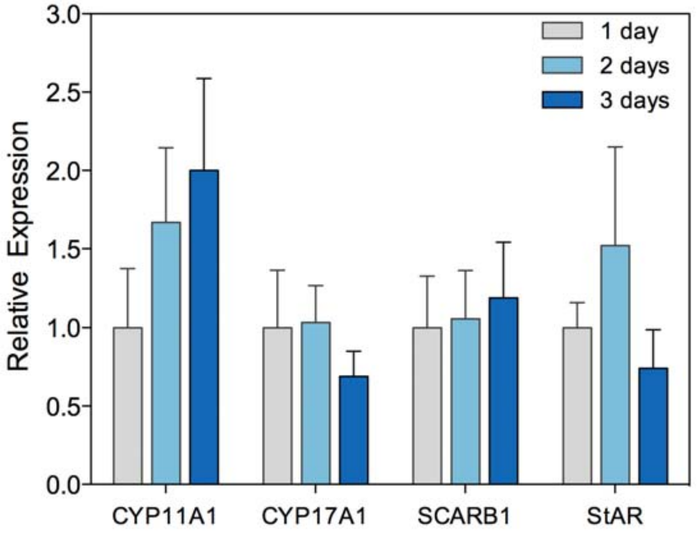

Supplemental Material, Figure S4. Steroidogenic gene expression in 6 control human testis into Nude rat xenografts after 1, 2, and 3 days of treatment. Values are mean expression ± SEM, relative to GAPDH, and normalized to day 1 expression. No significant differences were observed using a two-tailed t-test of 1d vs. 2d, 2d vs. 3d, or 1d vs 3d for any gene. Mean gestational age =19.16 weeks. These data are derived from a subset of samples presented in Figure 4, including only those 6 samples that were assayed on each of the 3 days.

Supplementary Material, Figure S5

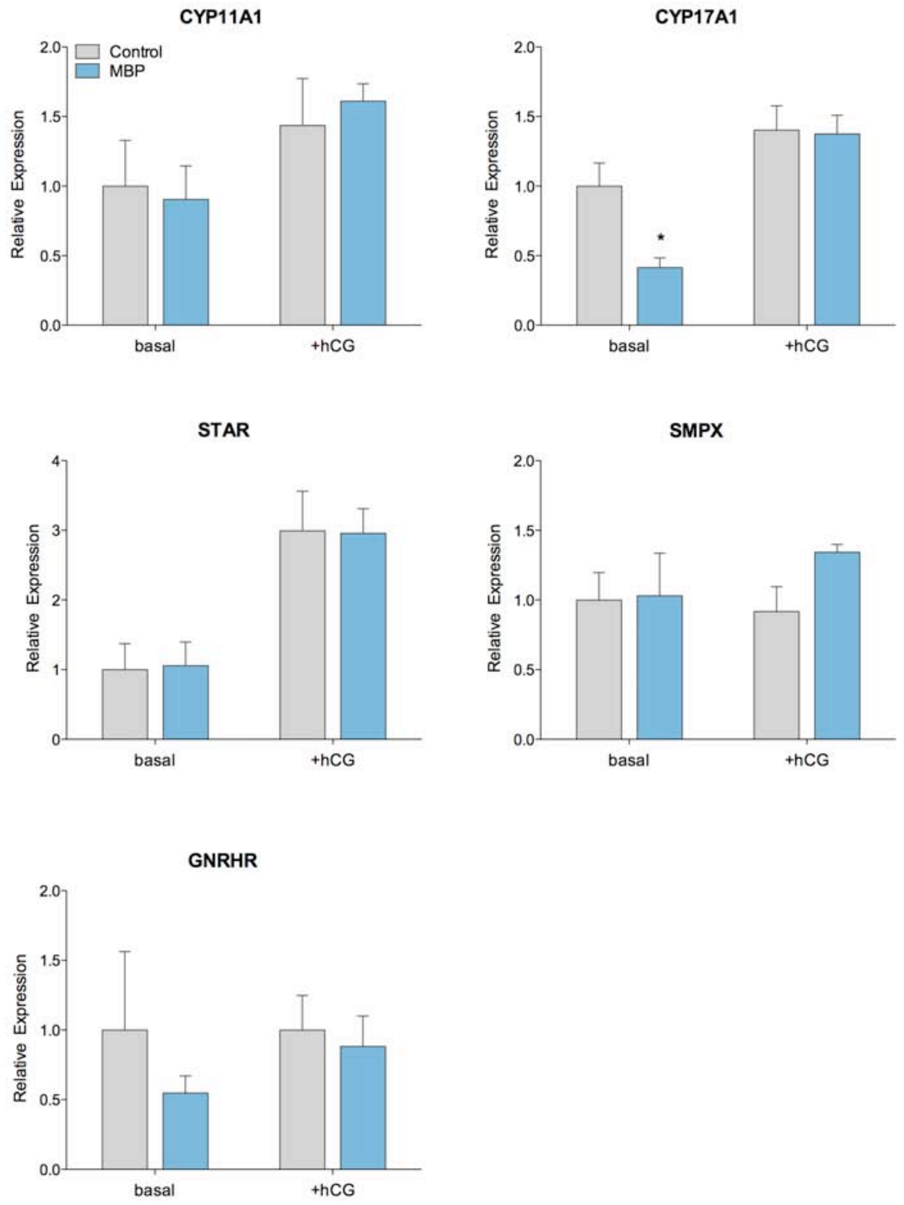

Supplemental Material, Figure S5. Gene expression of fetal rat testis cultured with monobutyl phthalate (MBP). Gd 17 testes were cultured for 24 hr in basal medium or medium containing 0.1 IU hCG without (control) or with 250 µM MBP. All values are mean expression ± SEM, relative to *Tbp*. N = 4 testes/group. **p* < 0.05 compared to medium without MBP by unpaired *t*-test.
